# Febrile illness management in children under five years of age: a qualitative pilot study on primary health care workers’ practices in Zanzibar

**DOI:** 10.1186/1475-2875-12-37

**Published:** 2013-01-28

**Authors:** Kimberly Baltzell, Kristina Elfving, Deler Shakely, Abdullah S Ali, Mwinyi Msellem, Shilpa Gulati, Andreas Mårtensson

**Affiliations:** 1 Departments of Family Health Care Nursing & Global Health Science, University of California San Francisco, San Francisco, CA, USA; 2 Malaria Research, Department of Medicine Solna, Retzius väg 10, Karolinska Institutet, Stockholm, 171 77, Sweden; 3 Department of Infectious Diseases, Institute of Biomedicine, University of Gothenburg, Gothenburg, Sweden; 4 Kungälvs Hospital, Department of Medicine, Kungälv, Sweden; 5 Zanzibar Malaria Control Programme, Ministry of Health, Mkwerekwe Street, Zanzibar; 6 University of Michigan School of Medicine, Ann Arbor, MI, USA; 7 Division of Global Health (IHCAR), Department of Public Health Sciences, Nobels väg 9, Karolinska Institutet, Stockholm, 171 77, Sweden

**Keywords:** Malaria elimination, Diagnostic decision-making, Non-malarial fevers

## Abstract

**Background:**

In Zanzibar, malaria prevalence dropped substantially in the last decade and presently most febrile patients seen in primary health care facilities (PHCF) test negative for malaria. The availability of rapid diagnostic tests (RDTs) allows rural health workers to reliably rule out malaria in fever patients. However, additional diagnostic tools to identify alternative fever causes are scarce, often leaving RDT-negative patients without a clear diagnosis and management plan. This pilot study aimed to explore health workers’ practices with febrile children and identify factors influencing their diagnostic and management decisions in non-malarial fever patients.

**Methods:**

Semi-structured key informant interviews were conducted with 12 health workers in six PHCFs in North A district, Zanzibar, April to June 2011. Interviews were coded using Atlas.ti to identify emerging themes that play a role in the diagnosis and management of febrile children.

**Results:**

The following themes were identified: 1) health workers use caregivers’ history of illness and RDT results for initial diagnostic and management decisions, but suggest caregivers need more education to prevent late presentation and poor health outcomes; 2) there is uncertainty regarding viral *versus* bacterial illness and health workers feel additional point-of-care diagnostic tests would help with differential diagnoses; 3) stock-outs of medications and limited caregivers’ resources are barriers to delivering good care; 4) training, short courses and participation in research as well as; 5) weather also influences diagnostic decision-making.

**Conclusions:**

This pilot study found that health workers in Zanzibar use caregiver history of fever and results of malaria RDTs to guide management of febrile children. However, since most febrile children test negative for malaria, health workers believe additional training and point-of-care tests would improve their ability to diagnose and manage non-malarial fevers. Educating caregivers on signs and symptoms of febrile illness, as well as the introduction of additional tests to differentiate between viral and bacterial illness, would be important steps to get children to PHCFs earlier and decrease unnecessary antibiotic prescribing without compromising patient safety. More research is needed to expand an understanding of what would improve fever management in other resource-limited settings with decreasing malaria.

## Background

Traditionally, malaria has been regarded as the most common and important febrile illness in sub-Saharan Africa [1]. With limited confirmatory diagnostic capabilities, the standard practice in rural primary health care facilities (PHCF), supported by past international guidelines, has been for all febrile illnesses to be managed presumptively as malaria [2]. In 2010, WHO changed its policy and now recommends parasitological confirmation before anti-malarial treatment is initiated [3]. In addition, malaria prevalence has decreased in several areas of sub-Saharan Africa in recent years [4] and consequently, most patients seen with febrile illness in these areas do not presently have malaria infection.

Zanzibar, an archipelago off of the Tanzania mainland, with a population of approximately 1.2 million [5], has been the site of a substantial joint effort in malaria control by the Zanzibar Ministry of Health and partners [6,7]. Available data suggest that the incidence of malaria has decreased significantly in recent years [6]. In fact, for every 100 febrile patients presently seen in PHCF in Zanzibar, approximately three will test positive for malaria [4, Shakely *et al*, unpublished data]. All PHCF in Zanzibar now have the tools (rapid diagnostic tests (RDTs) and/or microscopy) for confirmatory malaria diagnosis in febrile illness [8]. To improve management of fever patients with a negative malaria test, health care workers in resource-limited settings such as Zanzibar will require training and tools to offer alternative diagnoses and management [9]. *Plasmodium falciparum*-specific RDTs have generally shown a high sensitivity and specificity [10] in field studies and previous research indicates good adherence to RDT results among health care workers in Zanzibar [11]. This point-of-care test has given health care workers in Zanzibar an important tool to differentiate malaria from other febrile illnesses.

Misdiagnosing fevers may have important consequences. First, an incorrect malaria diagnosis leaves the patient vulnerable to worsening of the underlying true cause of fever [12]. Second, unnecessary anti-malarial treatment of non-malarial fevers with the generally recommended artemisinin-based combination therapy (ACT) is expensive [13] and could potentially spur evolution and spread of resistance towards these compounds, by exposing infecting parasites to sub-therapeutic doses of the long-acting partner drug during a considerable period of time [14-16]. Third, overuse of antibiotics could facilitate the selection of antibiotic resistance [17]. In this context, it is important to improve the understanding of what influences health workers’ diagnostic and treatment decision-making in febrile illness in Zanzibar [18].

The aim of this qualitative pilot study was to investigate primary health workers’ practices which lead to diagnostic and treatment decisions for febrile children under five years of age in Zanzibar rural health facilities and identify primary influences shaping clinical practice, including past training among health workers, types of diagnostic tools used, and educational factors.

## Methods

The study was conducted using semi-structured key informant (KI) interviews, a technique previously described and used to capture valid information about diagnostic decision-making in resource-limited settings [19-23].Twelve prescribing health workers (KIs) from all levels of health workers in Zanzibar who diagnose and treat patients were interviewed in person. Prescribing health workers included clinical officers, nurses, health orderlies and medical assistants. The study sites included six government PHCFs in Zanzibar North A district; Kivunge, Nungwe, Kijini, Chaani Kubwa, Tumbatu Gomani and Matemwe. Electronic thermometers, RDTs, stethoscopes, weight measures and tongue depressors were available at all study sites and microscopes were available at two of the six study sites.

Semi-structured, individual interviews lasting 60 min each, were conducted in the health facilities after office hours by two members of the study team (KE and DS). An English/Kiswahili translator was available during all interviews. The interview guide was translated from English to Kiswahili and then back-translated to ensure accuracy of the information. Questions were aimed at eliciting KIs’ personal experience and training in diagnosing and treating febrile children under five years of age. This population of patients was targeted as the study team conducted a concurrent study in the same area to identify the etiology of fever in children under five years of age who tested negative for malaria by RDT (Elfving et al, unpublished observations). Specifically, the questions included: work experience and previous training; the availability, use and perceptions of clinical diagnostic tools; and perceptions of and experience with febrile children. For example, one of the questions was: “*What are the most important things to look for when deciding how to diagnose a child who presents to the health facility with a fever?”* (Additional file 1).

### Ethical considerations

The study was approved by the University of California, San Francisco Committee for Human Research (UCSF-CHR) and the Zanzibar Medical Research Ethical Committee (ZAMREC). Key informants gave their written informed consent to have information regarding their diagnostic and treatment practices used in this analysis. All personal information that could identify the KIs was omitted from recordings, transcriptions and translations.

### Participants

Six PHCFs were purposively selected from North A district in consultation with the management of Zanzibar Malaria Control Programme and the district medical officer. Health facilities were chosen based on manpower capacity, i.e. at least two health workers available. Only one health worker was interviewed at a time to minimize interference with ordinary patient care. In each study site, two KIs were chosen based on their availability and willingness to participate. No health workers declined the offer to participate. All health workers received the cost of bus fare to and from the interview site. No other incentives were paid. The interviewers (KE and DS) had previously worked with nine of the KIs in another research study designed to evaluate the effectiveness of malaria RDTs in the hands of primary health care workers and their adherence to RDT results.

### Analysis

Interviews were audio-recorded and afterwards transcribed verbatim and translated into English by a bilingual (Swahili/English) speaking associate without any previous knowledge or relationship to the KIs. Interviews were uploaded into Atlas.ti [24] and coded to look for categories and major themes by two researchers independently using immersion/crystallization style of analysis [25]. Analysis was done by two members of the study team who did not conduct the actual interviews (KB and SG). In the event of differing interpretations, discussion between the researchers and verification with interviewers took place until consensus was reached. Data were analysed to provide a description of the site- and provider-level factors that play a role in the diagnosis and management of febrile patients. After initial analysis, the findings were validated by the interviewing researchers (KE and DS).

## Results

A total of six males and six females were interviewed, including two clinical officers, one public health nurse, five staff nurses, two health orderlies and two medical assistants. Their age and work experience ranged from 25 to 64 years and four months to 37 years, respectively. The following major themes were identified:

1. Health care workers use caregivers’ history of illness and results of malaria RDTs as the primary influence on initial diagnostic and management decisions. Health care workers value caregiver history but suggest caregivers need more education to avoid late presentation to health facilities and poor health outcomes;

2. There is uncertainty regarding viral *versus* bacterial illness and health care workers feel additional point-of-care diagnostic tests would help with differential diagnoses;

3. Stock-outs of medications (primarily antibiotics) and limited caregiver resources are listed as barriers to delivering good care and as contributing to poor outcomes;

4. Health care workers list training, short courses and participation in research studies as major influences on diagnostics;

5. Weather also plays a role in diagnostic decision-making.

Theme 1. Health workers use caregivers’ history of illness and results of malaria RDTs as the primary influences on initial diagnostic and management decisions. Health care workers value caregiver history but suggest caregivers need more education to avoid late presentation to health facilities and poor health outcomes.

In the absence of tests for differential diagnosis of febrile illness, this study found that health workers rely heavily on the history of the fever as told by the mother for diagnosing RDT negative children.

*“We usually ask the mother questions because we don’t have any other advance instruments or equipment to use.”* (C8)

*“We take history by asking mothers about diarrhoea, vomiting, convulsions, ear infection, coughing, fast breathing or normal breathing. Then we give diagnosis.”* (C11)

*“If the child is vomiting and has malaria symptoms but test negative then we ask the mother what other symptoms the child is suffering from.”* (C12)

Several health workers describe the importance of details about the fever, particularly if it has been intermittent or constant. An intermittent fever was associated with malaria and a constant fever with influenza or a wound infection. Cough is also reported as an important symptom to discuss with the caregiver, to help differentiate pneumonia from other illnesses.

*“The guardian can say the child has fever but you can ask them to tell you what kind of fever the child has, for example, my child is crying or is hot. You can ask how many days has your child had fever. With the guardian’s responses you can brainstorm and decide which investigation to carry out.”* (C3)

*“The most important thing is to know how many days the patient has had the fever. It is also important to know whether the fever has been constant or persistent. Also how the fever started. If the patient is coughing and has fever, we want to know which started first, fever or coughing. If coughing started first then that is the cause of the fever.”* (C1)

However, health workers noted that caregivers often believe that any fever is malaria and thereby characterized their perceptions of malaria as outdated. While the epidemiology of malaria has changed in Zanzibar over the last 10 years [26], caregivers still associate the majority of illness with fever, and fever with malaria. Health workers identified this lack of awareness as an impediment to adequately characterizing fever.

*“Every patient with fever imagines they have malaria… we try to advise that not every fever is malaria. We try to explain other causes of fever like tonsillitis, ear infection and urinary tract infection (UTI).”* (C1)

*“Most of the community members of [district] can’t differentiate which is fever because they think any condition they see, that even when someone has a wound, they say my child has fever.”* (C3)

RDTs for malaria were listed by all the KIs as the first test given to a child with fever. Some health workers cited examples of how they improved caregiver acceptance of RDTs by educating them at appointments and enforcing diagnostic results. Over time, caregivers began to seek out diagnostic testing when their child had fever. RDTs were believed to facilitate febrile illness management when caregivers have been educated to believe the results. These health workers emphasized that the involvement of key stakeholders on all levels of the health systems, the use of various media approaches, and frequent exposure, are key to creating this change in attitude.

*“Nowadays we don’t prescribe anti-malarials when one is RDT negative…When the child comes to the hospital we take history then we provide health education. We tell them there are a lot of things that cause fever so we have to carry out investigations to confirm malaria.”* (C3)

*“The patients don’t know the difference, homa is malaria and malaria is homa. If they have homa they ask for malaria medicine…We advise the patients to first take a test, and if they test positive they have malaria and if they test negative then they have fever caused by other diseases mentioned earlier or it could be flu.”* (C8) (Author comment: Homa is the word for fever in Kiswahili but has a wider meaning including diseases associated with fever or higher than normal body temperature.)

*“When we give health education frequently I think that can help us get the point we need…. When you give education to an individual there is change but it can’t happen in a few days.”* (C3)

*“Since they (RDTs) were introduced it has reduced malaria since a lot of people like coming to get tested. It has also made work easier.”* (C5)

Although health workers rely on information from the mother, health workers in this study also described the mother as a barrier to health care, particularly their role in impeding positive outcomes. When asked why patients continue to die in Zanzibar despite the progress in malaria reduction, the majority of health workers in this study mentioned mother’s delay in bringing the child to the health facility because they are distracted by work, other caregiving responsibilities, and financial constraints.

*“They are not treated early. Maybe the mothers delayed in bringing the children to hospital. So when they bring them the situation is severe and so when you treat it takes too long as they are so weak. Maybe they think that it’s just a fever because it is so cold, or they are too busy attending to family responsibilities, the father is away and left the mother with so many children and she has no time to get them to the hospital while others need to know the importance of health so that they can bring the children to hospital earlier. Most of the times we see the mothers are busy on the farm working, fishing, they do everything.”* (C2)

Several health workers suggested that educating the caregiver would improve health outcomes in children by teaching precautionary measures that result in fewer illnesses as well as early recognition of dangerous symptoms.

*“I think it is because the parents lack education. Parents need to tell the children not to play in the cold and wear warm clothing but sometimes the parents don’t follow instructions. Also kids play in dirty water and drink it so they get diarrhoea. We teach them about boiling water but the children are not always at home. When the children are playing outside the parents have no idea what they are doing but when they are indoors the parents will be able to watch and take care of them. I would make sure that there were enough…health education materials, especially for mothers in all facilities.”* (C9)

*“In our culture some people think that a child with severe anaemia is affected by devils so they delay to send to the hospital. If the child is sent to hospital early the health personnel can see that the child has severe [disease] and refer them for blood grouping and cross matching. This saves the life of the child.”* (C3)

*“Malaria has decreased but sometimes the problem is that mothers bring the children late to hospitals when the condition is already chronic.”* (C8)

Theme 2. There is uncertainty regarding viral versus bacterial illness and health workers feel additional point-of-care diagnostic tests would help with differential diagnoses

Many of the health workers interviewed were unclear about the difference between viral and bacterial infection, especially in the respiratory tract.

*“If the child has fever caused by infection, then we give antibiotics.”* (C8)

Health workers generally agreed on what should be treated with antibiotics. These conditions included pneumonia, tonsillitis, ear infections, other respiratory tract infections, boils, cellulitis, skin infections, and urinary tract infections. However, one clinician also listed chickenpox and measles (both viral childhood diseases) when asked which diagnoses require antibiotics.

*“We give antibiotics when they have pneumonia, ear discharge, gingivitis, and measles to reduce cough. Also,* (we give antibiotics for) *cellulitis, boils, abscess and chickenpox.”* (C5)

When asked what to prescribe for a child with a negative malaria test, several health workers listed antibiotics as treatment for general flu.

*“It depends on the situation. If the mother complains that the child is vomiting or has diarrhoea, worms, etc depending on the symptoms we prescribe drugs like mebendazole* (Author comment: mebendazole is an antiworm medication used in Zanzibar and other settings to treat hookworm, roundworm, pinworm and other worm infections) *or if it is flu we prescribe syrup or (antibiotic) depending on the child’s age.”* (C12)

*“For normal flu we give (antibiotic) syrup, if temperature is higher than 38.5 we prescribe paracetamol.”* (C8)

Health workers believe additional tools will increase their diagnostic accuracy, although they are unsure of which tools they prefer (tools to diagnose urinary tract infection and influenza are mentioned most frequently). Additionally, they reported a lack of adequate staff and laboratory tests as barriers to differentiating causes of febrile illness.

*“We use thermometers and stethoscope… we don’t have enough to check cultures, blood pictures, ESR.”* (C2)

*“I think every health centre needs to be better equipped and every laboratory should have a microscope. For example, children have anaemia but we don’t have the proper tools to determine that diagnosis.”* (C8)

*“There are a lot of other diseases like UTI infection we can’t diagnose because we don’t have investigation tool. We need laboratory equipment for UTI, Hb, and glucose because some come with hypoglycaemia… If a child has frequent fever, maybe no sign of malaria, test is negative and has no pneumonia, we need to check urine as a routine and for culture and sensitivity.”* (C11)

*“We have a laboratory that is not working as it has no personnel.”* (C7)

*“In my centre we got two great lab people but they were taken to another centre because we didn’t have the right equipment apart from a microscope.”* (C8)

Theme 3. Stock-outs of medications (primarily antibiotics) and limited caregivers’ resources are listed as barriers to delivering good care and as contributing to poor outcomes

Primary health care facilities in Zanzibar receive antibiotics and other medications on a quarterly schedule from a central supply group. Health workers in this study repeatedly identified stock-outs as an ongoing problem, suggesting that halfway through the quarter many commonly prescribed drugs were unavailable. Likewise, a limitation to prescribing medications was the inability of caregivers to pay for the medications.

*“Many patients are advised to buy medicine in private pharmacies. Some of them are available here for free but many of them are not available. Many of them are unable to buy the medicine. They return after two to three days without any medicine.”* (C1)

*“We get supplies but sometimes they are not enough to serve the children as the population [of district] has over 10,000 children. We can get syrup amoxicillin maybe four boxes of 24 so you can have (antibiotics) at the first of the month and none at the end of the month.”* (C3)

*“When the children come to the facility and we prescribe medicine they can’t afford the medicine. Sometimes the facilities don’t have enough drugs.”* (C11)

*“At the moment, many children are suffering from pneumonia so we prescribe medicine and then after a week they come with the same problem since some of them don’t buy the medicines.”* (C12)

*“In [district] there is a high illiteracy level… Also if they are transferred to (the local hospital) they can’t afford the transportation.”* (C12)

Theme 4. Health care workers list training, short courses and participation in research studies as major influences on diagnostics

In addition to formal training, past work experience and short courses such as the WHO Integrated Management of Childhood Illness (IMCI), RDT-training and research study training, seem to shape health workers' understanding of diagnostic tools and their clinical approach to febrile illness in children under five.

Educational background varied greatly among the health workers. Clinical officers had longer training periods and felt more confident giving diagnoses. When asked what the difference between health workers was, one clinical officer said:

*“I am better than others. (I am better) because of the course I took I am specially trained to review and diagnose the patients more closely so they recover quickly.”* (C10)

Health workers also report that working in private health facilities has influenced their thoughts on causes of febrile illness, as these health facilities often have more diagnostic tools and advanced practice health workers.

*“Also I am working in one dispensary in [district], this clinic is conducted by a paediatric consultant. He said that many patients admitted with fever, the main cause is urinary tract infection and not malaria. In this dispensary it is routine to do urinalysis in all patients and many of them it’s positive. When we give antibiotics the patients come back in two to three days in good condition.”* (C1)

The majority of the study participants have participated in IMCI training and past research studies. This subgroup recognized IMCI as a useful guide to differentiating diagnoses of febrile patients.

*“The training which gave me the knowledge and experience in febrile illness was the training on integrated management of childhood illnesses (IMCI).”* (C3)

Participation in research studies where advanced tools were available to confirm or refute health workers’ diagnoses was an eye-opener in alternative causes of febrile illness.

*“I was among the health workers who conducted RDT negative studies, the result hasn’t been sent back but I learnt a lot. There are some children we diagnosed as having common cold but when we sent to the laboratory the children had strep A positive* (Author comment: “strep-A” refers to Beta-Streptococci group A test from a throat swab). *IMCI says any fast breathing is pneumonia but when we did the RDT negative study we see few children with pneumococcal positive* (Author comment: “pneumococcal” refers to *Streptococcus pneumoniae* antigen test in urine). *We took urine culture during the study and some of the children had bacteria growth as sensitivity of the drugs. So the suggestion of any fever is malaria should be confirmed with diagnostic tools.”* (C3)

*“In RDT [negative] study we studied PCR, nasopharyngeal, streptococcus in urine. In normal conditions here this investigation is not done… We have no evidence to show that this (fever) is caused by bacteria. But according to signs and symptoms we can imagine it is caused by bacteria.”* (C1)

Health workers that had previously received RDT training were vocal about why they like RDTs.

*“RDTs are good and the results are rapid. It is also very simple to use and doesn’t take a lot of time.”* (C6)

*“They (RDTs) give accurate and timely results…especially the new type because you can know the type of parasite.”* (C9)

However, four of 12 health workers interviewed had not received training when they started using RDTs. Health workers who had not received RDT training at the time of interview stated that RDT negativity does not rule out malaria.

*“I know we use [RDT] to test for malaria but I don’t know the details… No, it’s not necessary [for RDT to be positive in diagnosing malaria].”* (C2)

*“RDT has uncertainties… People don’t believe the results.”* (C4)

Theme 5. Weather also plays a role in diagnostic decision-making

The majority of the health workers in this study used weather seasonality to explain changes in febrile illness incidence. Certain conditions were associated with either the rainy season or the dry season.

*“Febrile diseases usually occur in the rainy season when the body provides a mechanism to cope with the cold season. The summer is a normal season for them so the cold season is when they get fever.”* (C3)

When asked about the cause of fever in children:

*“Mostly it’s due to the weather especially when it is cold, children are coughing and have flu and fever.”* (C4)

*“During the rainy season we get a lot of diarrhoea cases and cholera. During the hot season we get flu because of the dust and conjunctivitis because of dust entering the eyes.”* (C5)

*“The rainy season is mostly associated with diarrhoea.”* (C8)

## Discussion

This is the first qualitative study to explore influences on prescribing health workers’ diagnostic and treatment decisions in febrile children under five years of age in the new context of low malaria transmission in Zanzibar. The study interviewers had previously worked with the majority of the study KIs in other research projects. These relationships, along with the use of semi-structured interview guides, were important in facilitating detailed discussion of what it is like to diagnose and manage febrile children in a setting where resources are limited. Given that the study KIs have likely had more training through participation in other research studies than the ordinary health worker in Zanzibar, these findings may potentially represent a better understanding of how to diagnosis and manage febrile children than would be found in health facilities which have not been involved in research activities. With this as background, there are five findings from this pilot study that could be useful in other settings in sub-Saharan Africa that recently have or presently are undergoing a rapid transition from high to low malaria transmission.

First, the results show that Zanzibar health workers’ usual initial diagnostic step when evaluating a febrile child, is the performance of a malaria RDT, followed by a detailed history from the caregiver. In particular, the KIs appear to trust malaria RDT results and were eager to explain their usefulness in the interviews. This is an important result since it supports the overall good health worker adherence to malaria RDT results previously reported in RDT effectiveness studies conducted in Zanzibar [11, Shakely *et al*, submitted]. Despite reliance on caregiver history of a child’s fever to guide diagnostic decisions, health workers pointed out that late presentation to health facilities is a major challenge in providing good care, and that education is essential to improve patient outcome. This delay in treatment seeking was assigned to several factors. Caregivers generally have multiple responsibilities, often multiple children to care for and limited resources for transport to health facilities. Additionally, caregivers may have to wait for long periods of time to be seen in the health facilities, perhaps making the health workers feel more compelled to give medication as opposed to suggesting a “wait and see” prescription. Given the important role caregivers play in diagnostic decision-making in many clinical settings, community educational programmes might target caregivers, primarily mothers, and should provide details of serious illness symptoms, when to seek care immediately and when to return to the health facility in the event the child does not improve.

Second, the KI interviews reveal that differentiating between bacterial *versus* viral causes of fever in children needs additional exploration and possibly the introduction of point-of-care diagnostics to guide treatment decisions. Although the KIs were not asked explicitly how to tell the difference between viral and bacterial causes of fever, many indicated that they prescribe antibiotics in either case. This has two important implications, one of which was highlighted in these interviews, namely, drug stock-outs. The other implication, which was not discussed explicitly during the interviews, is antibiotic resistance [27]. Access to antibiotics is crucial in a setting such as Zanzibar; however, drug stock-outs may be preventing access to antibiotics for those who truly need them. One health worker assumed that the patients’ failure to improve on antibiotics meant that they must have malaria instead. Also, in lengthy discussions with health workers regarding when they prescribed antibiotics, only one mentioned the need to look for antibiotic susceptibility to treatments prescribed. This suggests that the risk of development and spread of antibiotic resistance due to over-prescription of antibiotics often goes unconsidered. Some health workers cited a broad differential of diagnoses that they consider in diagnosing febrile children, but were concerned that their lack of diagnostic tools prevented them from proving or disproving their clinical suspicion.

Third, socio-economic barriers were underlying many of the study themes, such as medication stock-outs and lack of resources for caregivers to adhere to health worker recommendations. In resource-limited settings such as Zanzibar, the distribution of inadequate funds greatly impacts the quality of care received by patients visiting health facilities. Additional training and point-of-care diagnostic tests as suggested earlier may contribute to a more appropriate distribution of crucial medications, such as antibiotics. If health workers gain confidence in distinguishing between viral and bacterial illnesses, unnecessary prescribing of antibiotics may decrease and the supply of antibiotics may, in fact, be adequate for the population visiting the health facility. However, despite Zanzibar having a relatively well-developed infrastructure and easy access to primary health care facilities, transport for caregivers and their children to health facilities was highlighted as a barrier to adequate health-seeking behaviour. Additionally, if caregivers are sent to private facilities to purchase medications unavailable at government health facilities, it may adversely affect adherence to health workers’ recommendations.

Fourth, from this study, it appears that training, short courses on IMCI, and participation in research studies are fundamental to a health worker’s approach to febrile illness. The value of RDTs in health facilities and the accurate characterization of fever aetiology both hinge on health worker training in these domains. These results indicate that health workers who are trained in malaria RDTs trust the results and are therefore comfortable to educate the community on the usefulness of the tests. Additionally, health workers in this study felt confident after participating in research studies and observing tests that validate their diagnoses. Both these factors seem to have influenced and changed health workers’ diagnostic decision-making. Importantly, additional studies are needed to understand the relative importance of different aspects of health workers’ involvement in research activities and how that may influence their diagnostic decision-making. It is not clear whether the research training, supervision, previous exposure to additional diagnostic tools, or some combination of these factors produced the change in health workers’ attitudes and practices. All health facilities in this study had been part of past research studies and it appears that research studies have constituted a fair amount of ongoing education for health workers in the study area. However, is important to note that information from the study KIs may not represent the feelings of health workers in other PHCFs in Zanzibar that were not chosen to participate in research studies.

Finally, weather was mentioned by the majority of KIs in this study as a tool for guiding diagnostic decisions. The finding that fever is believed to be a mechanism to cope with rainy weather may prevent health workers in this setting from considering a broad array of diagnoses. It may, therefore, be useful in future health worker training to provide factual information on how the weather and seasons influence fever illness patterns.

Overall, KIs see their role as important as community educators and facilitators for improved health in their health facility catchment areas. In fact, health workers in these settings are considered crucial for reaching health-related Millennium Development Goals [28]. Because health workers are central to patient education, it may be an opportunity to more clearly integrate/involve them in dissemination of information to caregivers and families regarding malaria epidemiology and diagnostics, as well as alternative causes of fever. Likewise, following the clear message from these study health workers in the field, direct community education initiatives might be pursued to reinforce this approach, in order to impact community members on a broader scale.

The study has some limitations. First, it was a small pilot study, with only 12 KIs. However, health workers were included who represent all levels of health workers who staff Zanzibar government health facilities to address this issue and invite variety into perspectives offered. Additionally, all of the KIs worked in health facilities where previous research studies were conducted by the interviewers. This may have had the effect of introducing bias into the questions; however, interviews were coded and analysed by members of the study who had not previously worked in the study health facilities. Lastly, the majority of the KIs in this study have either participated in or worked in an environment where research on febrile illness in children has been conducted. This may have influenced their perceptions and diagnostic and treatment practices, limiting the ability to extrapolate the study findings to other settings where little research is being conducted.

## Conclusion

This pilot study found that health workers in Zanzibar use caregiver history of fever and results of malaria RDTs to guide management of febrile children. However, since most febrile children test negative for malaria, health workers believe additional training and point-of-care tests would improve their ability to diagnose and manage non-malarial fevers. Educating caregivers on signs and symptoms of febrile illness as well as introducing additional tests to differentiate between viral and bacterial illness would be important steps to get children to PHCFs earlier and decrease unnecessary antibiotic prescribing without compromising patient safety. More research is needed to expand understanding of what would improve fever management in other resource-limited settings with decreasing malaria.

## Abbreviations

ACT: Artemisinin combination therapy; Hb: Haemoglobin; IMCI: Integrated management of childhood illnesses; KI: Key informant; PCR: Polymerase chain reaction; PHCF: Primary health care facility; RDT: Rapid diagnostic test; UCSF-CHR: University of California San Francisco, committee for human research; UTI: Urinary tract infection; WHO: World health organization; ZAMREC: Zanzibar medical research ethical committee.

## Competing interests

The authors declare that they have no competing interests.

## Authors’ contributions

KB conceived of and designed the study and drafted the manuscript. KE and DS carried out the data collection in Zanzibar and contributed substantially to the design of the study and confirmed findings. MM and AA participated in the coordination of the study and the acquisition of data. SG performed data analysis and assisted with manuscript preparation. AM contributed to the study conception, the manuscript preparation and revisions. All authors read and approved the final manuscript.

## Supplementary Material

Additional file 1 Semi-structured interview guide. Click here for file
